# Exploration of Survival Traits, Probiotic Determinants, Host Interactions, and Functional Evolution of Bifidobacterial Genomes Using Comparative Genomics

**DOI:** 10.3390/genes9100477

**Published:** 2018-10-01

**Authors:** Vikas Sharma, Fauzul Mobeen, Tulika Prakash

**Affiliations:** School of Basic Sciences, Indian Institute of Technology Mandi, Kamand, Mandi, Himachal Pradesh 175005, India; vikas.sharma.biotech@gmail.com (V.S.); faizgwalior@gmail.com (F.M.)

**Keywords:** *Bifidobacterium*, comparative genomics, host-microbe interaction, human-gut, immunomodulations, niche-specific adaptations, pan-genome, probiotic

## Abstract

Members of the genus *Bifidobacterium* are found in a wide-range of habitats and are used as important probiotics. Thus, exploration of their functional traits at the genus level is of utmost significance. Besides, this genus has been demonstrated to exhibit an open pan-genome based on the limited number of genomes used in earlier studies. However, the number of genomes is a crucial factor for pan-genome calculations. We have analyzed the pan-genome of a comparatively larger dataset of 215 members of the genus *Bifidobacterium* belonging to different habitats, which revealed an open nature. The pan-genome for the 56 probiotic and human-gut strains of this genus, was also found to be open. The accessory- and unique-components of this pan-genome were found to be under the operation of Darwinian selection pressure. Further, their genome-size variation was predicted to be attributed to the abundance of certain functions carried by genomic islands, which are facilitated by insertion elements and prophages. In silico functional and host-microbe interaction analyses of their core-genome revealed significant genomic factors for niche-specific adaptations and probiotic traits. The core survival traits include stress tolerance, biofilm formation, nutrient transport, and Sec-secretion system, whereas the core probiotic traits are imparted by the factors involved in carbohydrate- and protein-metabolism and host-immunomodulations.

## 1. Introduction

Members of the genus *Bifidobacterium* are of immense significance because they have been identified as almost ubiquitous inhabitants of the human host and are known to exhibit probiotic effects, including anti-infectious and immunomodulatory activities. There are currently 74 recognized (sub)species of the genus *Bifidobacterium* (Table S1) as per the NCBI Taxonomy database as of July 2018 [1], which are found in a wide range of environmental habitats. Of these 74 (sub)species, at least nine have been found as the most abundant species in the human intestinal microbiome [2], including *B. bifidum*, *B. breve*, *B. longum*, *B. animalis*, *B. catenulatum*, *B. pseudocatenulatum*, *B. adolescentis*, *B. angulatum*, and *B. dentium*. Bifidobacterial species have been found to be involved in the biosynthesis of a wide-range of health-promoting active compounds, including short-chain fatty acids (SCFAs), vitamins, and organic acids [3]. In addition, these bacteria have also been demonstrated to modulate the host immune system by inducing immune-regulatory cytokines, including interleukin-6 (IL-6) and IL-8. Due to the professed beneficial health effects of these biomolecules, bifidobacteria have been exploited industrially for a long time as the potential probiotics.

Other than the probiotic features of bifidobacteria, their niche-specific adaptations are of great interest as these bacteria survive in the harsh environment of human gastrointestinal tract (GIT). Some bifidobacterial species have demonstrated the adoption of different strategies to cope with the gastrointestinal stress, including exposure to digestive enzymes, acidic pH, defensins, and antimicrobial peptides. For example, *B. infantis* Natren Life Start super strain (NLS-SS) has been found to reduce the expression of α-Defensin-5 in the mucosa of active celiac disease patients [4], *B. longum* biotype *longum* is demonstrated to control its intracellular pH for acid adaptation [5], and a few *B. bifidum* strains are found to be resistant to a potent antimicrobial peptide Lfcin [6]. *B. bifidum* is also found to produce extracellular sialidase, which enhances its adhesion to the mucosal surface and supports carbohydrate-assimilation [7]. In addition, some species of the genus *Bifidobacterium* have been demonstrated with acquired resistance to bile salt and antibiotics [3]. These studies demonstrate the promising potential of individual bifidobacterial strains for their survival- and probiotic-traits, particularly in human GIT. However, these functional potentials of many other bifidobacterial species and strains remain elusive.

There are few studies available which have attempted a comparative genomic analysis of the genus *Bifidobacterium* to explore its evolutionary conserved functional traits [8,9,10,11,12]. The core traits were found to be enriched in the functions related to housekeeping roles or those related to the adaptations to or interactions with a particular environment. To this end, carbohydrate metabolism, cell envelope biogenesis, amino acid biosynthesis and transport, and nucleotide biosynthesis and transport were among the highly abundant core functions, as reported in these studies. However, the bifidobacterial members included in these reports belong to a wide range of hosts and environments. Thus, they lack host-specific comprehensive functional analyses of the evolutionary conserved traits of bifidobacteria primarily in the gut environment. In addition, the possible interactions of these evolutionary conserved traits with the host genes and functions remained unexplored in these studies.

Being a significant probiotic and extreme survivor with characteristic small genomes, bifidobacterial genome evolution has been of great importance. Members of the genus *Bifidobacterium* demonstrate interspecific variations among their genome sizes, which reflect the differences among their metabolic capacities [8]. Thus, bifidobacteria might have been using genome evolution as a potential strategy to their advantage for niche-specific adaptations. Genome evolution, which is governed by the processes of horizontal gene transfer (HGT) and operation of selection pressure, lead to the events of gene-gain and gene-loss, which largely impact the gene-content of a given taxon. The gene content of bacteria can be used to define a given taxon and can be characterized by analyzing their pan-genome, which in turn is used to infer the genome evolution.

The pan-genomes at species level have been analyzed for a few bifidobacterial members in independent studies. The pan-genomes of most of the analyzed bifidobacterial species, including *B. breve* [13], *B. longum* [14], *B. bifidum* [15], *B. animalis* subsp. *lactis* [16], and *B. pseudocatenulatum* [17], were found to be closed. At the species level, only the members of *B. adolescentis* [18] were found to have an open pan-genome. In addition, a few interspecific studies have been carried out for bifidobacteria at the genus-level, albeit using a limited number of the members isolated from different habitats [8,9,10,11,12]. The pan-genome of the genus *Bifidobacterium* was found to be open in nature in these studies [8,9,11]. However, the number of genomes included in any study is a crucial factor for pan-genome calculations [19]. With the advancement in genome sequencing technologies, there has been a huge increment in the availability of sequenced bifidobacterial genomes. Thus, it is important to re-assess the nature of the pan-genome for the genus *Bifidobacterium* using all the available sequenced genomes in order to explore the bifidobacterial genome evolution.

The present study aims at performing a comprehensive comparative analysis of the genus *Bifidobacterium* to explore its genome evolution and functional aspects. In order to re-assess the genomic evolution of the genus *Bifidobacterium*, at first, we performed a pan-genome analysis by including the bifidobacterial species and strains from a wide range of environmental habitats. This was followed by a pan-genome calculation of only those members of the genus *Bifidobacterium*, which are found to inhabit the human GIT and are potential probiotics. The further analyses are focused only on the probiotic and human gut strains of bifidobacteria. For these members, we also explored the selection pressure operating on the bifidobacterial genomes to delineate its effect on the nature of the pan-genome. Further, the mobilome content of bifidobacteria is analyzed as a potential strategy for genome evolution. We have also performed a comprehensive functional evolution of the probiotic and human-gut strains of bifidobacteria (PHBifs) in order to explore the genome size variation as a potential strategy for niche-specific adaptations. The functional analysis is focused on a comprehensive exploration of the bifidobacterial molecular factors, which are related to their survival strategies in human GIT and probiotic-traits.

## 2. Materials and Methods

### 2.1. Retrieval of Genome Sequences

Initially, we have retrieved the 215 bifidobacterial genomes along with their detailed information (Assembly Level, Host Name, and Isolation Source) (Table S2), available at NCBI GenBank [1]. Of these, only those bifidobacteria were selected, which have completely sequenced genomes. These bifidobacteria are further classified into two groups, including those belonging to either human GIT or other origins. All bifidobacterial species/strains belonging to human GIT were selected for the analyses. Using an extensive literature survey, several of these bifidobacterial strains were found with probiotic potentials, whereas the probiotic status of a few other strains remained unknown. Among the bifidobacteria belonging to the origins other than human GIT, only those with known probiotic potential based on a thorough literature survey were added in the study. So in total, we used the 56 PHBifs for our analyses (Table 1). A flowchart of methodology and bioinformatics tools used for the present analysis is given in Figure S1.

### 2.2. Pan-Genome Analyses

Pan- and core-genome analyses were performed using BPGA v1.2 tool [41]. USEARCH v8.1.1861 [42], which is the default clustering algorithm of BPGA v1.2, was used for orthologous gene identification and clustering with a 50% sequence identity cut-off. Pan-genome plot was extracted by plotting the total number of distinct gene families identified with each subsequent addition of a genome against the number of genomes. Core-genome plot was extracted by plotting the total number of shared genes with the addition of each genome against the number of genomes. To avoid sampling biases, 30 iterations (random samples) were used for extracting these plots. Representative sequences of both pan- and core-genomes were used for comprehensive functional analyses.

### 2.3. Selection Pressure Analyses

Relative entropy was used as a quantification technique to delineate the effect of selection pressure on the pan-genome of PHBifs. To this end, we used a previously described method [43] for calculating the relative entropy of core and chromosome (whole genome) bases. In brief, the relative entropy was calculated in terms of the Kullback-Leibler Divergence (DKL) using the observed and expected frequencies of codons in coding regions. A low DKL value indicates a low relative entropy and a high DKL indicates a high relative entropy. Further, we employed the Welch’s *t*-test (*p*-value < 0.001) to estimate the statistical significance of difference between the relative entropies of the core and chromosome of PHBifs. All the statistical analyses were performed using R programming packages (https://www.R-project.org).

To unravel the nature of selection pressure, we have further calculated the selection pressure (ω) as a ratio of the non-synonymous (*K_a_*) to synonymous (*K_s_*) substitution rates. Towards this, we sequentially performed gene-wise alignments for all the core genes of PHBifs using ClustalW algorithm [44] within the MEGA-CC v7.0.14 [45] with the default parameters. These alignments were further used to calculate the *K_a_* and *K_s_* values using the Nei-Gojobori (NG) method [46] implemented in KaKs_Calculator v1.2 [47] with default parameters.

### 2.4. Genome-Size Variation and Mobilome Analyses

Clusters of orthologous groups of proteins (COGs) were inferred using the script CDD2COG.pl v0.1 [48]. Results were parsed using an E-value cut-off of 10^−4^, a threshold of 30% amino acid sequence identity, and 60% coverage of both the query and the hit in the alignment.

IslandViewer v4.0 [49] was used to predict the genomic islands (GIs) with default settings. IslandViewer 4 combines three different GI prediction methods: IslandPick [50], Score-based Identification of Genomic Islands-Hidden Markov Models (SIGI-HMM) [51], and IslandPath-DIMOB [52]. The predictions by SIGI-HMM were used for further analyses. In case of no result by SIGI-HMM, predictions by at least one method were used for the analyses. COGs functions were retrieved for GIs from the corresponding bifidobacterial genome annotations.

ISsaga v2.0 [53] and PHASTER [54] were used with the default parameters for the identification of insertion elements (IEs) and prophage sequences.

The Spearman’s R statistic was used to estimate the significant correlation between the two groups. The Kruskal-Wallis test was used to estimate the significant differences among the multiple groups. The results with a *p*-value < 0.001 were considered as statistically significant. All the statistical analyses were performed using the R software. GENE-E matrix visualization and analysis program [55] was used to create heatmaps.

### 2.5. Functional Analyses

For the functional analyses, *B. kashiwanohense* JCM 15439 was taken as a representative strain and its 613 core proteins were further subjected to a comprehensive functional analysis. For the comparative analyses, assignment of orthologs, and similarity searches, a threshold of 30% amino acid sequence identity, and 60% coverage of both the query and the hit in the alignments were used. In order to predict the orthologs, BLASTP v2.2.29+ [56] was used with an E-value cut-off of 10^−4^ and the best hits were selected.

Functional annotation of all the core protein sequences was done using COG module of WebMGA server [57] with an E-value cut-off of 10^−4^. Pathway mapping was done using BlastKOALA module of Kyoto Encyclopedia of Genes and Genomes (KEGG) online server [58] using prokaryotes as the taxonomy group and genus_prokaryotes as the KEGG genes database. Subcellular Localization Predictive System (CELLO) Server v2.5 [59] was used for the prediction of the subcellular-localization of these proteins (parameter used, organism: Gram positive). SecretomeP Server v2.0 [60] was used for the prediction of non-classically secreted proteins. Here, SecP score ≥ 0.5 was used as the determining value to indicate possible secretion. TMHMM Server v2.0 [61] was used for the prediction of transmembrane helices in proteins. LipoP Server v1.0 [62] was used for the prediction of lipoproteins and signal peptides. SignalP Server v4.1 [63] was used with the default parameters for the prediction of signal peptide cleavage sites in the amino acid sequences. Transporter proteins were predicted using Transporter Classification Database (TCDB) [64] by performing BLASTP (v2.2.29+) with an E-value cut-off of 10^−10^. To predict the carbohydrate active enzymes, the standalone version of Database for Automated Carbohydrate-Active Enzyme Annotation (dbCAN HMMs v5.0) [65] was used. The results were parsed with the following two parameters recommended for bacteria (i) if alignment > 80 amino acids, use an E-value < 10^−18^; otherwise use E-value < 10^−3^ and (ii) covered fraction of Hidden Markov Models (HMM) > 0.35. MvirDB database [66] was used for predicting the virulence factors using BLASTP (v2.2.29+) with an E-value cut-off of 1 × 10^−10^. To predict the secreted proteins and the protein secretion systems, EffectiveDB server [67] was used with enabled “genome mode” and prediction module “EffectiveS346”. The core proteins were further searched against Database of Essential Genes (DEG) v13.3 database [68] with the default parameters to identify essential proteins. For the identification and analysis of regulatory proteins, P2RP Server v2.7 [69] was used. MAHMI database [70] was used for the prediction of the sequence of potential immunomodulatory and antiproliferative peptides encrypted in the SecA protein sequences of 56 genomes of PHBifs.

### 2.6. Interspecific Interactions between Human and Bifidobacteria

The computational prediction of host-microbe protein-protein interactions (PPIs) was carried out through BIPS, that is, BIANA interolog prediction server [71] using the bifidobacterial core proteins (representative strain: *B. kashiwanohense* JCM 15439) as query against human database. The results were parsed by setting following homology conditions, Blast E-value: 1 × 10^−10^, Joint E-value: 1 × 10^−10^, % identities: 30, Joint identities (%): 30, Query sequence coverage (%): 60, and Template sequence coverage (%): 90. In addition, the template interactions were filtered by excluding the co-complex methods as tandem affinity purification.

Functional analyses of predicted human proteins were carried out using the annotations retrieved from UniProt [72]. Towards this, UniProt Entry of BIPS was used for screening the UniProt database. Further, these UniProt entries were subjected to screening for the human innate immunity-related proteins. In this direction, UniProt gene names were used for screening InnateDB database [73]. Co-occurring proteins (COP) were grouped into co-occurring protein function family and were excluded from the further analyses. NAViGaTOR v2.3 [74] was used for the graphical representation of the interactions.

For pathway analysis of unique human immunity-related proteins, their corresponding sequences were retrieved from UniProt database. These protein sequences were subjected to the metabolic pathway mapping with KEGG pathway database using KEGG Orthology and Links Annotation (BlastKOALA) program [58].

### 2.7. Interactome Analysis of Bifidobacterial Core Proteins

To evaluate the PPIs among the human interacting core proteins of PHBifs, STRING database [75] was used. The human interacting core proteins of *B. kashiwanohense* JCM 15439 were taken as the representative sequences for this analysis.

## 3. Results

### 3.1. Pan-Genome Analyses

**Figure 1:**
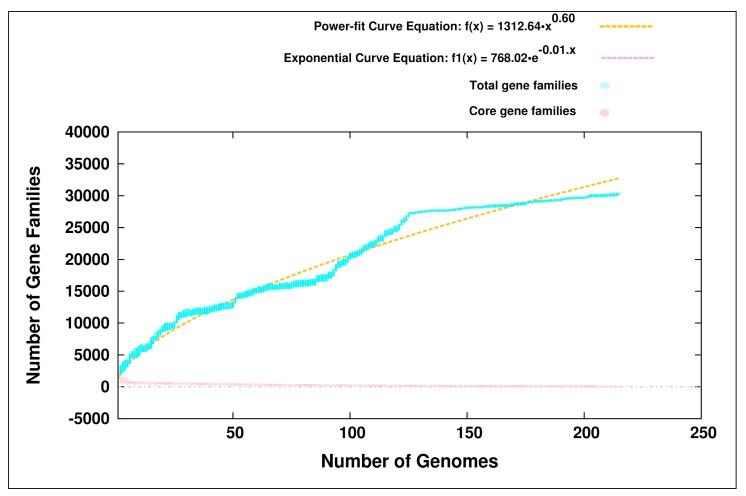
Plot of pan- and core-genomes of the genus *Bifidobacterium* (*n* = 215 members). Pan-genome estimate is shown after using 30 random samples of the 215 genomes. The plot represents a stabilized core structure but an open pan-genome.

We performed a pan-genome analysis of the genus *Bifidobacterium* by including the 215 members belonging to 44 different species inhabiting various hosts (Table S2). The pan-genome of the genus *Bifidobacterium* was predicted to be open in nature (Figure 1). This pan-genome was found to harbor 30,293 genes (Table S3), whereas the core-genome of the genus *Bifidobacterium* was found to be highly strict, comprising of only 12 housekeeping genes (Table S4). The pan-genome of a given taxon is affected by the complexity of the environmental conditions in which they are found to inhabit [76]. To this end, a pan-genome analysis was performed using the 56 members of PHBifs (Table 1). Interestingly, this pan-genome of the PHBifs was also found to be open in nature (Figure S2) and constituted 9772 genes (Table S5), including 613 conserved genes (PHBifsCore) (Table S6).

### 3.2. Selection Pressure Analyses

**Figure 2:**
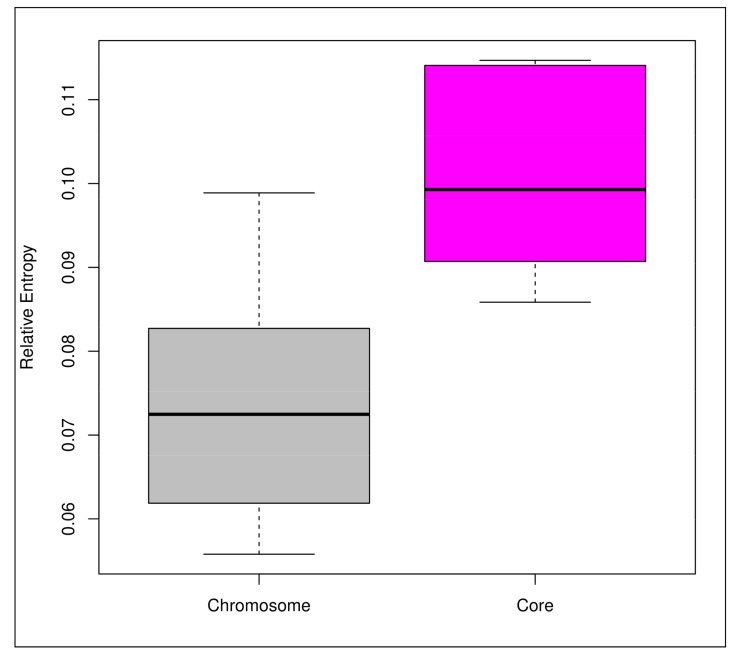
The relative entropy of chromosome versus core-genome for the 56 PHBifs. The relative entropy depicts the magnitude of the selection pressure. The core part has a higher selection pressure than that of the chromosome. Significant differences between the two groups were tested with the Welch’s *t*-test (*p*-value < 0.001).

**Figure 3:**
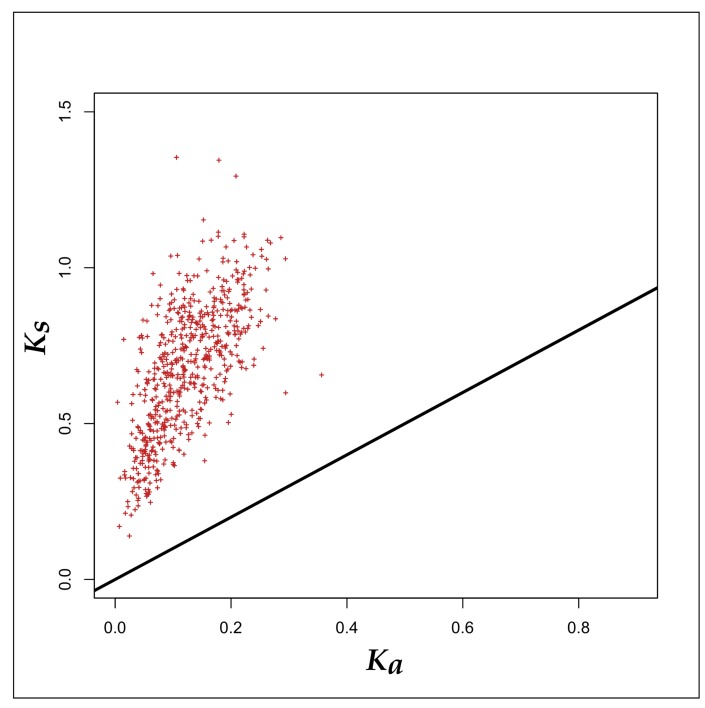
A plot of *K_a_* versus *K_s_* of the 613 gene groups of the orthologous genes from the 56 PHBifs. Straight line represents *K_a_* = *K_s_*. Each symbol (+) represents a gene group of 56 orthologous nucleotide sequences from the 56 bifidobacterial genomes. The figure implies that the rates of synonymous substitutions for 613 core genes is higher than that of the non-synonymous substitutions. The resulting core proteins will not be altered and remain conserved during evolution.

In order to determine the driving force behind an open pan-genome of the PHBifs, we calculated the selection pressure operating on the 56 genomes of PHBifs. Towards this, the levels of relative entropy for each of the PHBif genomes were calculated in their respective conserved core and chromosomal (whole genome) components. Comparison of these two groups using the Welch’s *t*-test indicated that the relative entropy in the bifidobacterial core-genomes was significantly higher (*p*-value < 0.001) than that of the corresponding whole genomes (Figure 2). However, the relative entropy only predicts the intensity of selection pressure irrespective of its nature (Darwinian and purifying). To unravel the nature of the selection pressure towards being favorable or against the amino acid changes in a given protein sequence, we have further calculated the selection pressure (ω) as a ratio of non-synonymous (*K_a_*) to synonymous (*K_s_*) substitution rates. The selection pressure (ω) for the core genomes of the PHBifs were evaluated by calculating the *K_a_*/*K_s_* ratios for all the 613 core genes individually. The ω-values for all the core genes were < 1 (average ω = 0.18). These observations point to a higher synonymous to non-synonymous mutation rate and indicate a prevalence of stringent purifying or stabilizing selection acting against amino acid changes in the core genes (Figure 3).

### 3.3. Mobilome Analyses

The genome sizes of the 56 PHBifs are found to vary greatly among themselves (Figure S3). Genome-size variation as a result of the genome evolution of a given taxon in a specific environment is significantly attributed to the horizontally transferred GIs. To this end, the GIs were predicted followed by their size- and abundance-estimations in the genomes of all the PHBifs (Table S7). The size and abundance of GIs reflect the bifidobacterial nucleotide content acquired by HGT and the frequency of HGT events, respectively. The bifidobacterial species having smaller genomes (*B. animalis*) were found to possess lesser abundance of GIs in comparison to the bifidobacterial species having comparatively larger genomes (*B. longum*). A correlation analysis revealed that the genome sizes of PHBifs had a very strong positive association with the sizes and abundances of their GIs (Table S8). Propagation of GIs is largely carried out by the mobilome (mobile genetic elements) of a given genome, which is essentially composed of IEs and prophages. Insertion elements and prophages were predicted for all the PHBifs (Table S7). The abundance of IEs was found to have a very strong positive correlation with the genome-size, GI-size, and GI-abundance. Further, the size and abundance of prophages were also predicted to have a moderate positive correlation with the genome-size and the GI-size.

### 3.4. Functional Analyses

**Figure 4:**
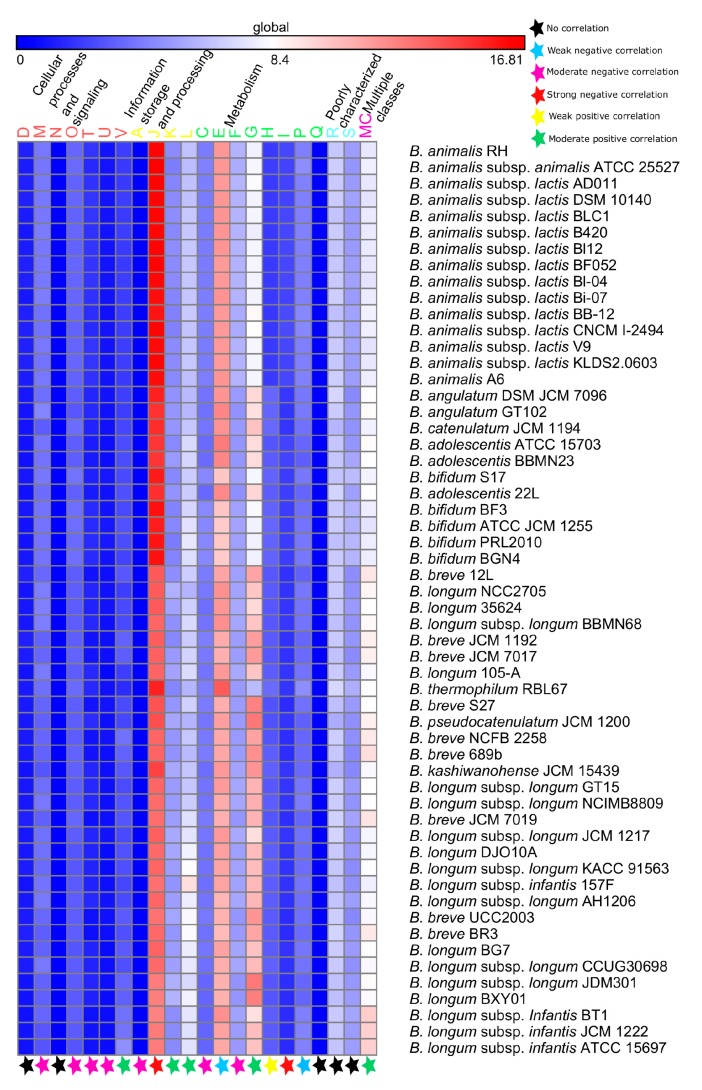
An abundance heat map of the different COGs classes present in the genomes of the 56 PHBifs. The strains are sorted (top to bottom) according to increasing genome sizes. D: Cell cycle control, cell division, chromosome partitioning; M: Cell motility; N: Cell wall/membrane/envelope biogenesis; O: Posttranslational modification, protein turnover, chaperones; T: Signal transduction mechanisms; U: Intracellular trafficking, secretion, and vesicular transport; V: Defense mechanisms; A: RNA processing and modification; J: Translation, ribosomal structure and biogenesis; K: Transcription; L: Replication, recombination and repair; C: Energy production and conversion; E: Amino acid transport and metabolism; F: Nucleotide transport and metabolism; G: Carbohydrate transport and metabolism; H: Coenzyme transport and metabolism; I: Lipid transport and metabolism; P: Inorganic ion transport and metabolism; Q: Secondary metabolites biosynthesis, transport and catabolism; R: General function prediction only; S: Function unknown; MC: Multiple classes. The Spearman’s R statistic (*p*-value < 0.001) was used to estimate the significant correlation between the two groups, including COGs class and genome-size, star represents the status of correlation; for details refer to Table S9.

The functional attributes of PHBifs are explored in order to gain an insight into the genome evolution, survival features, probiotic traits, and host interactions of bifidobacteria particularly in the human GIT. To explore the functional repertoire of PHBifs, their genomes were subjected to COGs functional analysis followed by the abundance estimation of COGs classes in all of them. These genomes were predicted to harbor the genes assigned to 21 COGs classes belonging to four COGs categories (Figure S4). In addition, the genes assigned to more than one COGs classes were grouped into “Multiple Classes (MC)”. The group “Multiple Classes (MC)” and the COGs categories “General Function Prediction Only (R)” and Function Unknown (S) were excluded from the further analyses. Among the 19 COGs classes, Translation, Ribosomal Structure and Biogenesis (J), “Amino Acid Transport and Metabolism (E)”, “Carbohydrate Transport and Metabolism (G)”, and “Replication, Recombination and Repair (L)” were among the most abundant functional COGs classes. Further, we carried out a correlation analysis for obtaining the significant associations between the abundances of COGs classes and the genome sizes of the PHBifs in order to explore which functions are increased or decreased with the variations in bifidobacterial genome sizes (Figure 4 and Table S9). Importantly, the abundances of “Carbohydrate Transport and Metabolism (G)”, “Defense Mechanisms (V)”, “Transcription (K)”, and “Replication, Recombination and Repair (L)” COGs classes were found to have a significant positive correlation (*p*-value < 0.001) with the genome sizes of bifidobacteria. In contrast, the abundances of “Amino Acid Transport and Metabolism (E)”, Translation, Ribosomal Structure and Biogenesis (J)”, “and “Cell Wall/Membrane/Envelope Biogenesis (M)” COGs classes were predicted to have a significant negative correlation (*p*-value < 0.001) with the genome sizes of bifidobacteria.

A gain of certain functions in bacterial genomes are facilitated by the accumulation of GIs in the genomes, which is governed by the process of HGT. In order to explore the trends in functions, which undergo HGT via GIs, the genes of predicted GIs of the PHBifs were subjected to COGs functional analysis. In each of these PHBifs, a majority of the horizontally transferred genes (average, ~86%) remained unassigned to any functional class. A further analysis of the assigned functional classes revealed “Replication, Recombination and Repair (L)” as the most abundant functional COGs class (Figures S5 and S6). In addition, the functions related to “Cell Wall/Membrane/Envelope Biogenesis (M)”, “Defense Mechanisms (V)”, and “Carbohydrate Transport and Metabolism (G)” COGs classes were also predicted to be enriched in the GIs of the PHBifs.

Horizontal gene transfer events impact the gene content of a given taxon in a given environment leading to genomic variations. Yet, there are certain conserved components of the genomes which are essential for the survival in a given niche. In order to get an estimation of this conserved part as well as that which may undergo changes as a result of the environmental demands, comprehensive functional analyses of the pan-genome of the PHBifs were carried out. At first, the pan-genome was subjected to KEGG pathway analysis. The accessory and unique components of the pan-genome were found to have “Membrane Transport”, “Carbohydrate Metabolism”, “Metabolism of Cofactors and Vitamins”, “Signal Transduction”, “Antibiotic Resistance”, “Amino Acid Metabolism”, and “Lipid Metabolism” among the most abundant pathways (Tables S10 and S11). “Nucleotide Metabolism”, “Translation”, “Replication and Repair”, and “Carbohydrate Metabolism” were among the most abundant pathways in the core component of the pan-genome.

The core-component of bacteria comprises the evolutionary conserved genes, which are essential for their survival and taxon-specific traits in a given environment. Thus, a comprehensive functional analyses of the 613 proteins of the PHBifsCore were carried out using a representative member of the PHBifs, viz., *B. kashiwanohense* JCM 15439, to explore their core genomic features, which are summarized in (Table 2 and Table S6). These proteins were classified into different functional classes using COGs. Out of the 613 proteins, 442 were mapped to 419 unique COGs, which were further classified into Metabolism (40.50%), Cellular Processes and Signaling (12.90%), Information Storage and Processing (30.54%), and Poorly Characterized (9.05%) functional COGs categories. The remaining 7.01% proteins were assigned to more than one COGs category and were grouped into “Multiple Classes”. In order to explore the pathways to which proteins of the PHBifsCore were mapping, the predicted proteins were annotated using KEGG database. A total of 488 proteins (79.61%) showed similarity with 471 unique KEGG identifiers corresponding to different metabolic pathways.

Besides the pathway enzymes, the gut bacteria uses a wide range of carrier proteins to transport specific molecules inside and outside the cells for their growth and survival in human GIT. Among the transported molecules, those with extracellular sub-cellular localizations are of great importance as the bacteria secrete or express these specific molecules outside their cells and some of these may participate in the host-microbe interactions. To this end, the 613 proteins of the PHBifsCore were subjected to “Transporter Proteins” and “Sub-cellular Localization” predictions. Sixty proteins were predicted as putative membrane transporters in the PHBifsCore. A further analysis classified the 613 proteins into four categories based on their subcellular localization, including cytoplasmic (81.08%), membrane (14.03%), extracellular (4.73%), and cell wall (0.16%). In addition, 94 non-extracellular proteins were predicted to be secreted non-classically. In total, 123 proteins were predicted to be secreted via either the classical or the non-classical pathways of secretion. Like most intestinal bacteria, bifidobacteria are saccharolytic and are involved in carbohydrate fermentation in the colon. In this direction, carbohydrate-active enzymes (CAZymes) analysis predicted 11 PHBifsCore proteins as the CAZymes involved in the transport and metabolism of carbohydrates. Further, we carried out comprehensive analyses of the significant core functions conserved in bifidobacteria at the genus level towards their adaptation-, survival-, and probiotic-traits in human GIT.

#### 3.4.1. Probiotic-Traits

Under the most abundant COGs category of “Metabolism” of the PHBifsCore, “Amino acid Transport and Metabolism (E)” (~12%) was its most abundant class, which was in corroboration with the other studies. A high abundance of the “Amino acid Transport and Metabolism (E)” genes in the PHBifsCore reflected the significance of amino acids for the growth of bifidobacteria in the human-gut environment. The dietary components of humans are rich in proteins, which upon microbial proteolysis produces free amino acids and nitrogen for both the host and microbes. Towards this, the PHBifsCore harbored several gene copies of the proteolytic enzymes, including peptidase, dipeptidase, aminopeptidase, and iminopeptidase. In addition, it also possessed a few amino acid carriers, including the aromatic and glutamate transporters. These proteolytic enzymes and transporters assist bifidobacteria by enhancing the fermentation capacity to produce the essential microbial growth factors, including amino acids and SCFAs [77,78,79]. The SCFAs exert multiple beneficial effects on the host energy metabolism and play a key role in the prevention and treatment of the metabolic syndrome, bowel disorders, and certain types of cancer. The PHBifsCore was also found to encode bile salt hydrolase, which is an enzyme produced by the intestinal microflora that catalyzes the deconjugation of glycine- or taurine-linked bile salts resulting in the release of free amino acids [80]. Bile salt hydrolase make the probiotics more tolerant to bile salts and helps to reduce the blood cholesterol level of the host which is a significant probiotic trait.

The “Carbohydrate Transport and Metabolism (G)” was the next abundant class (~7% of the PHBifsCore (*n* = 31)) under the “Metabolism” COGs category. It is known from earlier studies that over 8% of all the identified genes in the entire genome of most of the bifidobacterial strains are predicted to be involved in carbohydrate metabolism [81]. One of the major roles of the gut bifidobacteria is the degradation of complex carbohydrates into smaller units, which are further metabolized for deriving energy. For this purpose, they utilize a wide range of dietary carbohydrates, including plant-derived oligo- and poly-saccharides, which escape degradation in the upper parts of the intestine. These dietary carbohydrates may act as prebiotics to stimulate the selective growth of bifidobacteria. The proteins belonging to the “Carbohydrate Transport and Metabolism (G)” COGs function primarily encoded glycolytic and plant-derived polysaccharide and the related substrates (starch, maltodextrins) degrading enzymes. In addition, ~2% of the PHBifsCore (*n* = 11) was found to encode specific proteins related to the three classes of CAZymes, including glycoside hydrolase, glycosyl transferase, and carbohydrate-binding module. The former two enzymes are involved in the production of oligosaccharides from plant polysaccharides and milk sugar, whereas the latter is a catalytic domain present in the former two enzymes [82]. This domain particularly assists in the attachment of polysaccharide granules to the enzymes for the degradation of polysaccharides. For the degradation of plant-derived polysaccharides in the human GIT, bifidobacteria must secrete certain enzymes. To this end, alpha-xylosidase and isoamylase were found to be a part of the extracellular PHBifsCore with the former involved in the degradation of xyloglucan oligosaccharides and the latter involved in the hydrolysis of α-(1→6) bonds of amylopectin and dextrin.

In addition to dietary metabolism, the host-microbe interactions also impart significant beneficial effects, which include an enhanced colonization ability of the commensals via immunomodulations. These interactions mediate the probiotic-effects and have been reported, in a few cases [83], to be facilitated by the action of the proteins or peptides exported by bifidobacteria which are either secreted out of the cell or remain surface-exposed with their attachments to the bacterial cell wall or membrane. The extracellular proteins are also implicated in several other processes, including niche-specific adaptations, adhesion, nutrient uptake, and stress sensing. Towards this, we analyzed the extracellular proteins, including both the surface and the secreted proteins, in the PHBifsCore to look for the potential candidates primarily involved in the gut-associated immunity and adaptations. Among the 613 PHBifsCore proteins, 123 (~20%) were predicted as extracellular in nature. The extracellular PHBifsCore included immunomodulatory proteins viz., Deoxyribonuclease and molecular chaperone DnaJ. The deoxyribonuclease enzyme from *B. longum* BB536 [83] is involved in the biogenesis of immunostimulatory oligodeoxynucleotides (ISS-ODNs), which are known to suppress IgE production and Th2 immune responses. Similarly, the bacterial DnaJ protein is found to induce pro-inflammatory cytokine production in macrophages through the PI3K/JNK signaling pathway [84]. It also inhibits the proliferation of autoreactive T cells and induces the expression of IL-10 in the patients with rheumatoid arthritis [85]. The extracellular PHBifsCore was also found to harbor von Willebrand A (VWA) domain-containing proteins, which are known to interact with the epithelial extracellular matrix [86]. In addition, the PHBifsCore also harbored pilus assembly protein, which is known to elicit immune response by increasing TNF-α production [83]. In addition to these extracellular factors, a recent study demonstrated the role of protein translocase subunit SecA in triggering Th17 immune response via an encrypted immunomodulatory peptide (“FAIVDEVDSILIDEAR”). To this end, we mined the conserved SecA protein sequences of all the PHBifs for this encrypted immunomodulatory peptide. “FAIVDEVDSILIDEAR” was found to be the conserved SecA immune eliciting peptide in the proteomes of three bifidobacterial strains, including *B. kashiwanohense* JCM 15439, *B. catenulatum* DSM 16992, and *B. pseudocatenulatum* DSM 20438, whereas the proteomes of the remaining 53 species and strains harbored its variant viz., “YAIVDEVDSILIDEAR”, as the conserved SecA peptide with a predicted potential of anti-inflammation.

#### 3.4.2. Survival-Strategies

The next abundant COGs category of “Information Storage and Processing” largely included “Translation, Ribosomal Structure and Biogenesis (J)”, “Replication, Recombination and Repair (L)”, and “Transcription (K)” COGs classes, which are implicated in the basic cellular functions. The “Cellular Processes and Signaling” COGs category of PHBifsCore primarily includes “Defense Mechanisms” COGs class to combat against the environmental stress. The PHBifsCore harbored genes are implicated in multiple acid resistance (Table S6) to maintain a pH homeostasis. In addition, the proteins involved in the translocation of various ions were also found in the PHBifsCore, which are essential to maintain the inorganic ions homeostasis in bacteria via different import or export mechanisms. A high abundance of the toxic metal ions and an extremely acidic pH level of the gut environment makes it a harsh ecological niche for bacteria. In order to survive in such an environment, the gut bacteria adopt certain stress-coping adaptive strategies, including sodium/proton antiporter for pH hemostasis and uptake and efflux mechanisms for metal ion homeostasis. For heat- and osmotic-tolerance in the gut environment, the PHBifsCore was found to have a few caseinolytic proteases (Clp), which are known to have specific role to cope with these stressful conditions in bifidobacteria [87].

Approximately 10% of the PHBifsCore was comprised of the carrier proteins, including ATP-binding cassette (ABC) transporters, glutamate transporters (GTs), and phosphate transport system (PTS) related proteins. These transporters are primarily associated with the nutrient uptake in bacterial systems. The transport systems and facilitators are one of the important factors for the bacterial survival and growth in a given system. Their presence in the PHBifsCore suggests their conserved nature as an adaptation strategy for the acquisition of key nutrients and metabolic intermediates essential for bifidobacterial growth and survival in human GIT. In addition, these transporters have been found to be involved in several additional functions too. For example, ABC transporters function in drug resistance [88], whereas GTs and PTS are strongly associated with acid tolerance [89] and phosphate homeostasis [90], respectively.

Besides transporters, secretion systems constitute a significant component of bacterial transport systems and have been demonstrated to play significant roles in niche-specific adaptations [91]. To this end, the PHBifsCore encoded the essential components of the general secretory pathway (Sec pathway) viz., SecA, SecE, SecG, and SecY, which function in exporting the unfolded proteins in bacteria [92]. In addition, the accessory components of the Sec machinery including ftsY, GroEL, and GroES, were also found to be present in the PHBifsCore. Interestingly, no other secretion pathway was found to be conserved in the PHBifsCore. These observations suggest that the probiotic and human gut strains of bifidobacteria use Sec pathway as a housekeeping function for the secretion of the important proteins as an adaptation strategy in the complex environment of human GIT.

Bifidobacteria are known to exhibit biofilm formation [3], which is another significant feature of the gut colonizing bacteria and is required for their survival in host GIT via efficient polysaccharide metabolism and antibiotic resistance. Towards this, the PHBifsCore harbored several factors involved in the biogenesis of biofilm. For example, it possessed Lon protease, which is implicated in the motility and biofilm formation in a mucin-rich environment [93]. It was also found to encode S-ribosylhomocysteine lyase (LuxS), which helps in the biofilm formation via the production of the interspecific signaling molecule autoinducer-2 (AI-2) [94]. Similarly, N-glycosyltransferase was another significant component of the PHBifsCore which is involved in the biogenesis of biofilm. These observations suggest the biofilm as a significant and evolutionary conserved feature of the PHBifs. In addition, the PHBifsCore was also found to encode several extracellular ribosomal proteins (Table S6). This is in corroboration with the previous studies, which found the ribosomal proteins as extracellular (surface-associated) components [60]. Besides their regular functions, the ribosomal proteins could have extra ribosomal functions, including moonlighting and regulatory roles [95]. The moonlighting is a phenomenon in which a protein performs multiple, apparently unrelated, jobs in the economy of the cell. Thus, the ribosomal proteins may adapt to the specific environments and lifestyles of the living species [96], which might impart assistance to bacteria in niche-specific adaptations.

### 3.5. In Silico Protein-Protein Interaction Analyses

The coevolution of the hosts with their microbes involves highly sophisticated interactions, which might impart beneficial traits to both the host and the microbes for their survival and growth. To this end, we carried out an in silico host-commensal interactome analysis using the PHBifsCore and human proteome. Only 34 PHBifsCore proteins were predicted to be interacting with 3181 human proteins leading to 6836 interactions (Tables S12 and S13). Among these 3181 human proteins, 1964 were assigned to 487 protein families. Actin, annexin, tubulin, MHC class I, and heat shock protein 70 were among the most abundant protein families, constituting ~26% of the assigned UniProt protein families. Among the bifidobacterial interacting proteins, “Posttranslational Modification, Protein Turnover, Chaperones” was the most abundant COGs functional class (~49%) contributed to by dnaK, groL, pepO, and dnaJ proteins. Besides interacting with the human proteins, the microbial proteins also interact with each other. The hubs of such PPIs indicate the essentiality of these proteins to participate in the critical interactions of the biological processes [97]. In order to explore the interactions of the 34 PHBifsCore proteins with each other, their protein interactome analysis was also carried out using an in silico approach. Among these, 31 proteins were found to be interacting with each other, whereas the remaining proteins, including rmlB, pepO, and pepC, were predicted to be non-interacting (Table S14).

**Figure 5:**
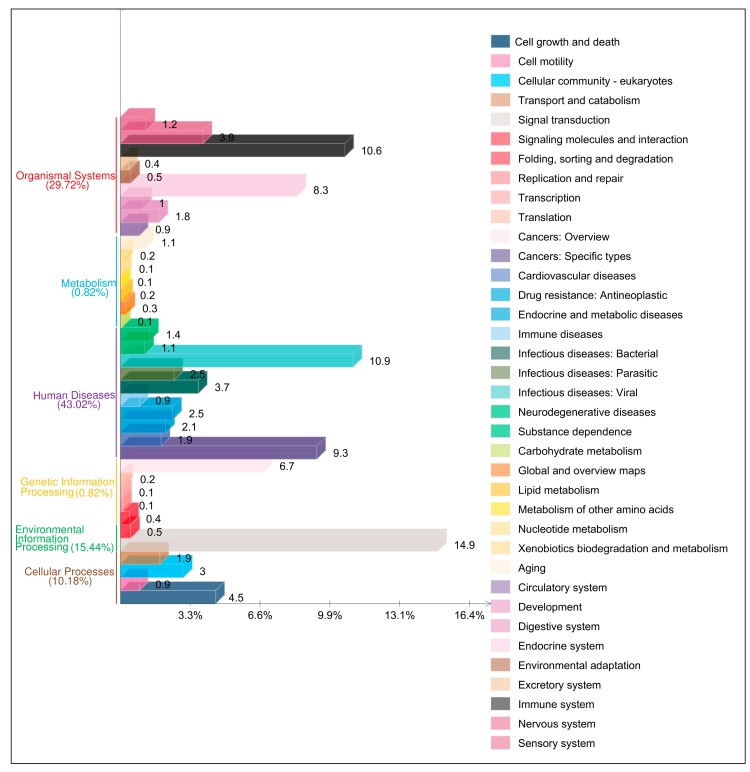
A bar plot of the KEGG pathways of the human innate immunity-related proteins, which are interacting with the core proteins of the 56 PHBifs. The pathway legends corresponding to the bars (from bottom to top) are given on the right panel.

The bifidobacterial members are known to play important roles in immunomodulatory functions in the host. Thus, we explored the interactions of the PHBifsCore proteins with human innate immunity-related proteins. Only 19 PHBifsCore proteins were found to be interacting with 289 innate immunity-related UniProt proteins and their variants leading to 439 interactions (Table S15). Among these human innate immunity-related proteins, peptide antigen binding (~30%), transferase activity (~12%), protein binding (~9%), and phosphoprotein binding (~5%) were the most abundant functions. These 289 UniProt proteins and their variants belonged to 109 human innate immunity-related unique functional proteins. The PPI-interaction network of these proteins and 34 PHBifsCore proteins is given in Figure S7. A KEGG pathway analysis of these 109 human innate immunity-related proteins assigned ~84% of these proteins with KEGG IDs corresponding to different pathways (Table S16). Of these pathways, “Human Diseases (~43%)” was found as the most abundant pathway category with “Infectious Diseases: Viral” as its most abundant sub-category (Figure 5 and Table S16). “Organismal Systems (30%)”, “Environmental Information Processing (~15%)” and “Cellular Processes (~10%)” were the next abundant pathway categories with “Immune System”, “Signal Transduction”, and “Cell Growth and Death” as their most abundant sub-categories, respectively. The predicted interacting signaling pathways under “Infectious Diseases: Viral” category primarily include Ras, MAPK, PI3K-Akt, cAMP, Rap1, ErbB, Jak-STAT, Sphingolipid, TNF, FoxO, and Wnt signaling pathways. Among the bifidobacterial interacting proteins, “Posttranslational Modification, Protein Turnover, Chaperones” was the most abundant (56%) COGs functional category contributed by dnaK, groL, and pepO proteins. In addition, gap and pgk belonging to the COGs functional category “Carbohydrate Transport and Metabolism” were among the other significant bifidobacterial proteins predicted to be interacting with the human host immune system.

## 4. Discussion

### 4.1. Open Pan-Genomes of the Genus Bifidobacterium and its Probiotic and Human-Gut Strains

In order to gain a better understanding of the complex ecology of bifidobacteria, it is important to analyze their genomic evolutionary traits, which make them susceptible to the heritable genetic variations that can arise from the adaptations in response to the environmental changes. The genomic evolutionary aspects of bacteria are explored by analyzing their pan-genome, which is bound to their lifestyle. The pan-genome includes a core-genome containing the genes shared by all the strains of the genus under consideration, a dispensable genome containing the accessory genes present in two or more, but not in all the strains, and the genes which are unique to the individual strains. The pan-genome is used to infer the evolutionary status of a give taxon in terms of its open or closed nature. An open pan-genome reflects the capability of a given taxon to undergo the events leading to gene-gain and -loss, whereas a closed pan-genome indicates a saturated gene-content which is less likely to acquire any additional functionalities [8].

However, the pan-genome is greatly affected by the numbers of genomes included in the analysis. It is important to note that, Bottacini et al., 2010 [9], Sun et al., 2015 [11], and Milani et al., 2014 [8] considered 14, 45, and 47 members of the genus *Bifidobacterium*, respectively, which inhabit various hosts and sources and predicted an open pan-genome. Interestingly, even after increasing the number of genomes (*n* = 215) in our analysis by at least five-fold as compared to the previous studies, the pan-genome of the genus *Bifidobacterium* remained open. This open pan-genome indicated a continuous expansion of this genus, which could be attributed to the different evolutionary mechanisms, including HGT, speciation, diversification, and evolutionary selection pressure operating on the genomes. The primary driving forces behind the genus expansion are the events of gain and loss of functions, which imparts with the microbes an enhanced ability to adapt to a wide range of ecological niches and to respond to the precipitous environmental changes [98]. To this end, the 215 members of the genus *Bifidobacterium* were found to inhabit a wide range of environmental habitats (Table S2), thereby exhibiting significant inter-species variations leading to niche-specific genomic adaptations. This was in corroboration with the recent studies, which demonstrated that the pan-genome of the microbes found in the diverse environments is open in nature [99].

Another factor which is known to affect the nature of the pan-genome is the complexity of the specific environment in which the bacteria inhabit [98]. Towards this, we observed an open pan-genome of those members of the genus *Bifidobacterium*, which are limited to the human GIT and are potential probiotics. This indicates a continuous evolution of these bifidobacteria in the human GIT, which may be attributed to the complexity of this environment. The open pan-genome of the PHBifs indicates an accumulation of changes in their genomes, primarily due to the Darwinian (positive) selection pressure. However, it does not reveal whether these changes are being accumulated in the core or the accessory component of the pan-genome or both. In addition, the intensity of the Darwinian selection pressure remains unknown. We observed a substantially stronger selection pressure operating on the core-genome component in comparison to the non-core part of the bifidobacterial genomes. The conserved core genes of the PHBifs are crucial for the growth and survival in a given ecological niche and the operation of strict purifying selection pressure on these genes confirmed their critical roles in bifidobacterial growth and survival in a given environment. The prediction of a stringent stabilizing selection pressure on the core-genome also discards the possibility of the accumulation of new functionalities in the core-component of the pan-genome. This suggests that the non-core components of the pan-genome, including the accessory and/or unique genomes, may be the major sites for accumulating the changes (non-synonymous mutations) leading to the evolution of new genes and taxa. Thus, these non-core genes may be primarily responsible for an open pan-genome of the PHBifs.

These accumulated changes at the protein level may primarily confer specialized functions for niche-specific adaptations to the bifidobacteria in human GIT. An adaptive evolution induced by the accumulation of new traits leads to the genome-size variation of a given taxon in a specific environment. To this end, bifidobacteria exhibits characteristically small genome-sizes, albeit with significant variations. For example, one species of this genus, *B. indicum* LMG 11587, is 1.73 Mb in genome size whereas, another species, *B. biavatii* DSM 23969, is almost two-fold larger (3.25 Mb) [8]. The genome size variations among the bifidobacterial genomes reflect their adaptive evolution and might have arisen primarily due to the horizontally transferred GIs, which impart with these bacteria the accessory functions for selective advantages, for example, for niche-specific adaptations and antibiotic resistance. In contrast, the core component of the pan-genome constitutes the evolutionary conserved functions, which are essential for the growth and survival of the bacteria in a given environment.

### 4.2. Survival- and Probiotic-Traits of the Probiotic and Human-Gut Strains of Bifidobacteria

Being probiotic bacteria and having human GIT as a natural habitat, bifidobacteria possess certain functions as an evolutionary conserved component of their genomes which might be of immense significance to themselves as well as their hosts. Although there are several functional studies available, they are limited to the functional profiling of a particular species or strain of bifidobacteria [3,4,5,6,7,14,15,16,17,18,100]. Only a limited number of studies are available at the genus level [8,9,10,11,12], which nevertheless lack detailed functional analyses. To this end, our study provides a comprehensive view of the core functions conserved in bifidobacteria at the genus level towards their survival and probiotic traits in the human GIT. The GIT represents one of the largest interfaces between the host, environmental factors, and antigens in the human body and harbors ~10^14^ microorganisms. The harsh environment of the human GIT makes it difficult for the survival of most microorganisms. Bacteria adopt certain survival strategies to cope with this environmental stress of human GIT.

For microorganisms, the human GIT is a very stressful environment being highly acidic and flooded with heavy metal ions [101]. As a stress-coping strategy, the genes for pH-, ionic/osmotic-homeostasis were found to be conserved in the PHBifs, which are essential to survive in human GIT. These genes primarily include transporters, which are involved in the movement of ions, small molecules, and macromolecules, across the biological membranes. Besides the transporters, the conservation of Sec secretion system only, among all the known bacterial secretion systems, highlights its importance for the bifidobacterial survival in the competitive environment of human GIT. The Sec secretion system is known to be involved in the secretion of many vital proteins as well as some toxins and additional virulence factors [102]. The Sec secretion system may also aid in the colonization of commensals via transporting the specific substrates [103]. Interestingly, this conserved secretion system of the probiotics and human-gut strains of bifidobacteria also possesses the immunomodulatory functions as reported in the proteome of *B. longum* DJO10A in an earlier study [83], which may impart bifidobacteria with both the probiotic as well as the survival potential in the human GIT.

The gut microbes also develop the biofilms to colonize the human GIT. A biofilm is a complex and self-produced polymeric matrix-based site, where microorganisms can adhere to each other as well as to the mucosal surface and protect themselves from the environmental stress induced primarily due to the bile salts, acids, antimicrobial agents, and antibiotics [3]. In the biofilms, the bacterial cells can communicate with each other for the biofilm maturation and regulation of the degradative enzymes synthesis in response to the environmental stress [104]. Bifidobacteria are known to develop biofilm or micro colonies in the human GIT [94]. In fact, the PHBifs exhibit conservation of this function also during evolution and use it as one of its survival strategies as reported earlier for the bifidobacterial members belonging to a wide range of hosts and environments [12].

Besides these functions, replication, transcription, and translation-related functions are important to maintain the physiological parameters during the changes to which gut bacteria are suddenly exposed, thus suggesting their survival and adaptive roles [105]. A conservation of the “Translation, Ribosomal Structure and Biogenesis (J)”, “Transcription (K)”, and “Replication, Recombination and Repair (L)” COGs classes in the core-genome of the PHBifs depicts the significance of information storage and processing for these bacteria to survive and adapt to the complex environment of human GIT. In addition, the commensal gut bacteria use various defense mechanisms, including production of bacteriocins, regulation of host inflammation responses, and induction of the host immune system against pathogens [106]. Presence of the “Defense Mechanisms (V)” COGs class in the core-genome of PHBifs indicates the evolutionary conservation of the protective mechanisms in bifidobacteria in human GIT. Particularly, bifidobacteria uses the efflux pumps and carrier proteins to combat against the antibiotic-load in human GIT. Such transporter proteins are reported to be involved in the multidrug resistance mechanisms in bifidobacteria in earlier studies [107].

Towards the symbiotic relationship with host, probiotic bifidobacteria produce active compounds, which are beneficial to the host. For example, SCFAs, oligosaccharides, and unconjugated or deconjugated bile acids produced by bifidobacteria are important biomolecules, which impact the human physiology [108]. Evolutionary conservation of the functions responsible for the metabolism of these biomolecules in the core-genome of the probiotic and human gut-strains of bifidobacteria in our analysis is consistent with a previous study, involving bifidobacterial strains from a wide range of hosts and environments [12]. Besides these active compounds, bifidobacteria, just like other commensals also conserve the extracellular immunomodulatory proteins, which play a significant role in their interaction with the host [83]. These molecular interactions are the basis of the mutual relationship that bifidobacteria hold with their hosts.

### 4.3. Conserved Protein-Protein Interactions of the Human Host and the Probiotic and Human-Gut Strains of Bifidobacteria

The host-microbe interactions help bacteria in their survival and adaptation in the complex environment of human GIT [109]. It imparts the beneficial effects to the host as well. It is previously known that the host cellular membrane and cytoskeleton function as the significant physical sites for the host-microbe interactions [110]. Our predicted interactions supports this notion with a high prevalence of actin, annexin, tubulin, MHC class I, and heat shock protein 70 families among the human interacting proteins. The MHC class I complexes are exported to the plasma membrane surface via the Golgi apparatus where they are able to prime the CD8^+^ T-lymphocytes [111]. The actin, annexin, and tubulin proteins are primarily involved in the cytoskeleton assembly and membrane organization and trafficking [112,113]. Similarly, the interactions of bifidobacterial chaperones with the human proteins suggests the modulating potential of bifidobacteria towards human proteins. In this direction, a few bacterial chaperones have already been demonstrated to differentially modulate the human protein (α-Synuclein amyloid) formation via transient contacts [114]. Besides the interspecific interactions, bacterial proteins are also involved in the cross-talk among themselves leading to significant modifications [115]. Our analysis also found few human interacting bifidobacterial proteins, which may cross talk with each other as well as with the host system, thus depicting their significant roles. These PPI hubs can be used as the therapeutic targets for desired immunomodulations as demonstrated in previous studies [116,117]. Probiotics, synbiotics, and drugs can be used as the therapeutic molecules to induce these proteins to change their expression, which in turn may be exploited to modify their cross talk with the human system as a part of the predictive, preventive, and personalized medicine.

Towards host-microbe interaction, the innate immune system is a significant component, which elicits the pro-inflammatory responses and regulates the adaptive immune cells in response to the shift in microbial population and dynamics [118]. In this direction, a high abundance of the bifidobacteria interacting human innate immunity-related proteins in the “Infectious Diseases: Viral” pathways was observed. It suggests a regulatory function of these pathways as they are involved in several cell signaling processes implicated in the viral pathogenies. These signaling processes are pivotal for the multi-cellular organisms, as cells need to communicate extensively among each other and with the environment in order to coordinate actions for proper functioning and the well-being of the organism against the viral infection. The predicted interacting signaling pathways in the humans primarily include Ras, MAPK, PI3K-Akt, cAMP, Rap1, ErbB, Jak-STAT, Sphingolipid, TNF, FoxO, and Wnt, which have been demonstrated to play significant roles in the viral pathogenesis in several studies [119,120,121,122,123,124,125,126,127,128,129]. The highest interactions of bifidobacterial proteins with these signaling processes of “Infectious Diseases: Viral” pathways suggest a significant bifidobacterial role in the pathogenies and prognosis of the viral infections. However, it is important to note that these signaling pathways are also involved in several other functions and diseases. Our analysis further predicted the interactions of the human innate immunity-related proteins primarily with the bifidobacterial chaperones, endopeptidases, and glycolytic enzymes depicting their immunomodulatory roles. It is in corroboration with the earlier studies [130,131,132,133,134], which demonstrated host immunomodulatory functions of these proteins.

### 4.4. Functional Evolution Versus Genome-Size Variations among the Probiotic and Human-Gut Strains of Bifidobacteria

The accumulation of new genes in the accessory and unique components of the pan-genome is an evolutionary adaptive trait, which expands the genomic potential of a given microorganism for selective advantages. Addition of new functions for the adaptive purposes leads to the genome-size variations among the bacterial strains of a given environment. A correlation of the functional classes with the bacterial genome-sizes was found to be statistically significant for the bacterial genomes in previous studies [135]. The gain of functions is attributed to the GIs governed by the process of HGT. Thus, the functional classes, which are positively correlated with the genome-sizes are expected to be more abundant in the predicted GIs, whereas those functional classes, which are negatively correlated with the genome-sizes are expected to be less abundant in the predicted GIs. A significant correlation exists between the functional abundances and the genome-sizes of the PHBifs, which leads to the variations among their genome sizes. These genome size variations arise as a result of the HGT of GIs carrying these functions. Such events are well-known in the bacterial genomes, where some genes increase in response to the sudden demand of a given environment, whereas few other genes remain the same in the numbers to meet up with the demand attributed to the increase in gene numbers/genome-size of the bacteria as demonstrated in a previous study [135].

A positive correlation of the abundances of the “Replication, Recombination and Repair (L)” functional class is observed with the genome-sizes and this is also the highly abundant functional class in the genomes and GIs of the PHBifs. However, it is in contrast with the previous studies, which reported a negative correlation of this functional class with the genome size of bacteria including a few members of the genus *Bifidobacterium* [135]. The genes of this functional class are primarily involved in the establishment of the basic and essential cell structures and are reluctant to HGT. However, this functional class also includes the transposases, integrases, phage genes, which are highly prone to HGT. To this end, previous studies have reported high rates of HGT for the DNA modification protein encoding genes, which are important for the recombination and directly or indirectly promote the integration of foreign DNA in the genome [106]. The GIs impart a selective advantage to the recipient bacteria to adapt in a given environment. Thus, the HGT of the genes of “Replication, Recombination and Repair (L)” functional class in the PHBifs reflect the niche adaptations as demonstrated in recent studies [136,137,138].

A positive correlation of the abundances of the “Carbohydrate Transport and Metabolism (G)” with the genome sizes, and a high abundance of this functional class in the genomes and GIs of the PHBifs suggest their genome-size variations as a consequence of the acquisition of these functions via HGT for their adaptive evolution in human GIT. Horizontal gene transfer of the genes belonging to this functional class has already been reported in the genomes of *Escherichia coli* [137]. This functional class is essential for bifidobacteria to adapt to the saccharide-rich environment of human GIT as reported in earlier studies [81,83].

A negative correlation of the abundances of the “Translation, Ribosomal Structure and Biogenesis (J)” functional class is observed with the genome sizes of the PHBifs. This is in corroboration with an earlier study performed on several bacterial species, including bifidobacterial strains [135]. It suggests that a similar number of the given genes is able to cope with the elevated demand attributed to an increased number of genes in a given environment. The abundance of this functional class is found to be low in the predicted GIs in the PHBifs. Though, the HGT of the genes belonging to this functional class has been demonstrated as a part of the bacterial genome evolution for niche-specific adaptations [138,139].

A negative correlation of the abundances of the “Amino Acid Transport and Metabolism (E)” functional class with the genome sizes of the PHBifs corroborate with the low abundance of this class in the GIs, despite its high abundance in the bifidobacterial genomes. Nevertheless, this functional class is also implicated in the niche-specific roles as reported earlier [140]. The “Amino Acid Transport and Metabolism (E)” functional class primarily includes the biosynthesis as well as the catabolism of the amino acids. An acquisition of lesser number of genes by the PHBifs may be attributed to their auxotrophic nature, wherein these bacteria are not able to synthesize certain amino acids, instead they acquire them from the host [141]. Thus, bifidobacteria might not require full machinery for amino acid metabolism in human GIT, indicating that an addition of more genes belonging to this functional class may not be required by the probiotic and human gut strains of bifidobacteria.

Our analyses further suggest that the HGT of these genes is primarily carried out by the prophages and IEs, which regulate the frequency and size of GIs. The abundance and size of GIs have a positive correlation with the genome sizes of bacteria, as reported in earlier studies [142]. Thus, a direct association between the genome size and the abundance of the mobilome content can be inferred in terms of the interspecific variations among the genome sizes of the bifidobacteria in human GIT. This is consistent with previous studies, which reported an abundance increment of IEs and prophages with the bacterial genome sizes [143,144].

## 5. Conclusions

Comparative analysis of the publicly available 215 bifidobacterial genomes belonging to a broad range of niches allowed the description of the pan-genome of the genus *Bifidobacterium*, which was shown to follow an essentially open trend suggesting the futuristic expansion of this genus with novel species and strains. Interestingly, the pan-genome for the 56 bifidobacteria in a narrow niche of human GIT was also found to be open, which reveals the continuous evolution of these bacteria possibly with the novel traits specific to human GIT and probiotic functionalities. The evolution of novel traits is primarily attributed to the selection pressure, which was found to accumulate non-synonymous changes exclusively in the accessory and unique components of the pan-genome. The probability of gaining accessory functionalities by bifidobacteria is further strengthened by the positive correlation of their genome-size and mobilome-content, including IEs and prophages. This mobilome-content facilitates the HGT of GIs carrying certain functions, which add up to the genomes and leads to the variations among the PHBifs. Further, comprehensive functional analyses of the evolutionary conserved core-genome of these bifidobacteria enabled us to inspect it for the primary factors involved in the survival strategies and probiotic traits. Towards the survival strategies, bifidobacterial core-genome was found to have conserved functions related to the stress tolerance against acids and antibiotics, biofilm formation for adherence and defense, nutrient transport for cellular homeostasis, and Sec secretion system for the transport of cellular proteins. For the probiotic traits, the nutrient metabolism was found to be a characteristic feature of these bacteria having a wide range of proteolytic and saccharolytic enzymes as an integrated component of their core-genome. The immunomodulatory feature, imparted by bifidobacterial factors for host-commensal cross talk, was another significant probiotic-trait, which was found to be evolutionary conserved in the core-genome. The findings of the present study will help to widen our understanding of one of the most important probiotic microorganisms, which is of immense industrial value. This study is also anticipated to aid future investigations focusing on the probiotic design and personalized medicine.

## Figures and Tables

**Table 1:** List of the 56 probiotic and human-gut strains of bifidobacteria (PHBifs) used for the analyses.

Assembly	Organism/Name	Strain	Isolation Source	Probiotic Potential [Reference]
GCA_000695895.1	*B. animalis* RH	RH	Feces *	Yes [20]
GCA_000260715.1	*B. animalis* subsp. *animalis* ATCC 25527	ATCC 25527	Sewage	Yes [21]
GCA_000021425.1	*B. animalis* subsp. *lactis* AD011	AD011	Infant fecal sample *	Yes [3]
GCA_000022965.1	*B. animalis* subsp. *lactis* DSM 10140	DSM 10140	Commercially available probiotic strain	Yes [3]
GCA_000224965.2	*B. animalis* subsp. *lactis* BLC1	BLC1	Commercially available probiotic strain	Yes [3]
GCA_000277325.1	*B. animalis* subsp. *lactis* B420	B420	Commercially available probiotic strain	Yes [22]
GCA_000414215.1	*B. animalis* subsp. *lactis* Bl12	Bl12	Colonoscopic sample *	No
GCA_000818055.1	*B. animalis* subsp. *lactis* BF052	BF052	Feces of breast-fed infant *	Yes [23]
GCA_000022705.1	*B. animalis* subsp. *lactis* Bl-04	Bl-04; ATCC SD5219	Fecal sample from a healthy adult *	Yes [3]
GCA_000277345.1	*B. animalis* subsp. *lactis* Bi-07	Bi-07	Commercially available probiotic strain	Yes [22]
GCA_000025245.1	*B. animalis* subsp. *lactis* BB-12	BB-12	Commercially available probiotic strain	Yes [3]
GCA_000220885.1	*B. animalis* subsp. *lactis* CNCM I-2494	CNCM I-2494	Commercially available probiotic strain	Yes [24]
GCA_000092765.1	*B. animalis* subsp. *lactis* V9	V9	Feces of healthy Mongolian infants *	Yes [3]
GCA_000816205.1	*B. animalis* subsp. *lactis* KLDS2.0603	KLDS2.0603	Adult feces *	Yes [25]
GCA_000817045.1	*B. animalis* A6	A6	Feces *	Yes [26]
GCA_001025155.1	*B. angulatum* DSM 20098 = JCM 7096	JCM 7096	Human feces *	Yes [27]
GCA_000966445.2	*B. angulatum* GT102	GT102	Feces *	No
GCA_001025195.1	*B. catenulatum* DSM 16992 = JCM 1194 = LMG 11043	JCM 1194	Human feces *	Yes [3]
GCA_000010425.1	*B. adolescentis* ATCC 15703	ATCC 15703	Human adult intestine *	Yes [3]
GCA_000817995.1	*B. adolescentis* BBMN23	BBMN23	Human feces *	Yes [28]
GCA_000164965.1	*B. bifidum* S17	S17	Feces of a breast-fed infant *	Yes [3]
GCA_000737885.1	*B. adolescentis* 22L	22L	Milk *	Yes [29]
GCA_001281345.1	*B. bifidum* BF3	BF3	Feces *	Yes [30]
GCA_001025135.1	*B. bifidum* ATCC 29521 = JCM 1255 = DSM 20456	JCM 1255	Stool of breast-fed infant *	Yes [31]
GCA_000165905.1	*B. bifidum* PRL2010	PRL2010	Infant stool samples *	Yes [3]
GCA_000265095.1	*B. bifidum* BGN4	BGN4	Human feces *	Yes [32]
GCA_000568955.1	*B. breve* 12L	12L	Human milk *	No
GCA_000007525.1	*B. longum* NCC2705	NCC2705	Infant feces *	Yes [3]
GCA_001719085.1	*B. longum* 35624	35624	Ileal mucosa of an individual free of gastrointestinal disease *	Yes [33]
GCA_000166315.1	*B. longum* subsp. *longum* BBMN68	BBMN68	Long-lived man’s intestinal tract *	Yes [3]
GCA_001025175.1	*B. breve* DSM 20213 = JCM 1192	JCM 1192	Infant feces *	Yes [3]
GCA_000568975.1	*B. breve* JCM 7017	JCM 7017	Infant feces *	No
GCA_000829295.1	*B. longum* 105-A	105-A	Human feces *	Yes [34]
GCA_000347695.1	*B. thermophilum* RBL67	RBL67	Baby feces *	Yes [3]
GCA_000569075.1	*B. breve* S27	S27	Infant feces *	No
GCA_001025215.1	*B. pseudocatenulatum* DSM 20438 = JCM 1200 = LMG 10505	JCM 1200	Infant feces *	Yes [3]
GCA_000569035.1	*B. breve* NCFB 2258	NCFB 2258	Infant feces *	Yes [35]
GCA_000569055.1	*B. breve* 689b	689b	Infant feces *	No
GCA_001042615.1	*B. kashiwanohense* JCM 15439 = DSM 21854	JCM 15439	Feces of a healthy Japanese infant *	No
GCA_000772485.1	*B. longum* subsp. *longum* GT15	GT15	The gastrointestinal tract (GIT) of a healthy adult from Central region of Russia *	Yes [36]
GCA_001446255.1	*B. longum* subsp. *longum* NCIMB8809	NCIMB8809	Stool sample *	Yes [5]
GCA_000569015.1	*B. breve* JCM 7019	JCM 7019	Adult feces *	No
GCA_000196555.1	*B. longum* subsp. *longum* JCM 1217	JCM 1217	Intestine of adult *	Yes [3]
GCA_000008945.1	*B. longum* DJO10A	DJO10A	Healthy young adult’s feces *	Yes [3]
GCA_000219455.1	*B. longum* subsp. *longum* KACC 91563	KACC 91563	Feces of neonates *	Yes [3]
GCA_000196575.1	*B. longum* subsp. *infantis* 157F	157F	Human infant feces *	Yes [3]
GCA_001725985.1	*B. longum* subsp. *longum* AH1206	AH1206	Stool sample *	Yes [37]
GCA_000220135.1	*B. breve* UCC2003	UCC2003	Infant nursing stool *	Yes [3]
GCA_001281425.1	*B. breve* BR3	BR3	Feces *	Yes [38]
GCA_001293145.1	*B. longum* BG7	BG7	Feces *	Yes [39]
GCA_001446275.1	*B. longum* subsp. *longum* CCUG30698	CCUG30698	Human adult intestine *	No
GCA_000092325.1	*B. longum* subsp. *longum* JDM301	JDM301	Human infant feces *	Yes [3]
GCA_000730205.1	*B. longum* BXY01	BXY01	Gut *	No
GCA_001281305.1	*B. longum* subsp. *Infantis* BT1	BT1	Feces *	No
GCA_000269965.1	*B. longum* subsp. *infantis* ATCC 15697 = JCM 1222 = DSM 20088	JCM 1222	Intestine of infant *	Yes [40]
GCA_000020425.1	*B. longum* subsp. *infantis* ATCC 15697 = JCM 1222 = DSM 20088	ATCC 15697	Human infant feces *	Yes [40]

* Represents that isolation source belongs to the human host.

**Table 2:** Summary of the genomic and functional features of the core-genome of the 56 PHBifs.

Feature	Feature Count *	Sub-Feature	Sub Feature Count #
Protein Encoding Genes (PEGs)	613 (100%)		
PEGs predicted with the COGs functions	442 (72.1%)	Cellular Processes and Signaling	57 (9.3%)
		Information Storage and Processing	135 (22.02%)
		Metabolism	179 (29.2%)
		Multiple Classes	31 (5.06%)
		Poorly Characterized	40 (6.53%)
PEGs mapped to the KEGG functions	488 (79.61%)		
PEGs assigned to the Transporter Proteins	60 (9.79%)		
PEGs assigned to the Virulence Factors	118 (19.25%)		
Subcellular Localization of PEGs	613 (100%)	Cell Wall	1 (0.16%)
		Cytoplasmic	497 (81.08%)
		Extracellular	29 (4.73%)
		Membrane	86 (14.03%)
PEGs predicted with the Transmembrane Helices	78 (12.72%)		
PEGs predicted with the Signal Peptide Cleavage Sites	9 (1.47%)		
PEGs predicted with the Lipoprotein Signal Peptides	613 (100%)	Cytoplasmic Proteins	561 (91.52%)
		SPaseI-cleaved Proteins	12 (1.96%)
		Lipoproteins (SPaseII-cleaved Proteins)	1 (0.16%)
		Transmembrane Proteins	39 (6.36%)
PEGs predicted with the Non-Classical (Not Signal Peptide Triggered) Secretion	94 (15.33%)		
PEGs assigned to the Effector Proteins	69 (11.26%)	Endoplasmic Reticulum as an Effector Target	34 (5.55%)
		Mitochondrion as an Effector Target	7 (1.14%)
		Endoplasmic Reticulum as a Possible Effector Target	9 (1.47%)
		Mitochondrion as a Possible Effector Target	19 (3.1%)
PEGs assigned to the Essential Genes	496 (80.91%)		
PEGs assigned to the Types of Other DNA-binding Proteins	1 (0.16%)		
PEGs assigned to the Types of Transcription Factors	19 (3.1%)		
PEGs assigned to the Types of Two-Component Systems	7 (1.14%)		
PEGs assigned to the Carbohydrate Active Enzymes	11 (1.8%)	Carbohydrate-Binding Modules	2 (0.32%)
		Glycoside Hydrolases	6 (0.97%)
		Glycosyl Transferases	3 (0.48%)

*, # are calculated out of 613 PEGs. KEGG: Kyoto Encyclopedia of Genes and Genomes.

## Supplementary Materials

The following are available online at http://www.mdpi.com/2073-4425/9/10/477/s1, Figure S1: Flowchart of the methodology and the bioinformatics tools used for the analyses; Figure S2: A plot of the pan- and core-genomes of the 56 probiotic and human-gut strains of bifidobacteria (PHBifs) with complete genomes; Figure S3: A bar-plot of the variations among the genome-sizes of the 56 probiotic and human-gut strains of bifidobacteria (PHBifs); Figure S4: The abundance box plots of the different COGs classes present in the 56 probiotic and human-gut strains of bifidobacterial (PHBifs) genomes; Figure S5: The abundance box plots of the different COGs classes present in the GIs of the 56 probiotic and human-gut strains of bifidobacteria (PHBifs); Figure S6: An abundance heat map of the different COGs classes present in the GIs of the 56 probiotic and human-gut strains of bifidobacteria (PHBifs); Figure S7: An interaction network of the human innate immunity related proteins and the core proteins of the 56 probiotic and human-gut strains of bifidobacteria (PHBifs); Table S1: A list of the 74 recognized bifidobacterial species available at the NCBI Taxonomy database; Table S2: A list of the 215 members of the genus *Bifidobacterium* with publically available genomes (complete and draft); Table S3: The power and the exponential fit law implemented in BPGA pipeline to find out the estimated size of the core-genome and the open/close nature of the pan-genome for the 215 members of the genus *Bifidobacterium*; Table S4: A list of the genes (gene IDs of *B. kashiwanohense* JCM 15439) present in the core-genome of the 215 members of the genus *Bifidobacterium*; Table S5: The power and the exponential fit law implemented in BPGA pipeline to find out the estimated size of the core-genome and the open/close nature of the pan-genome for the 56 probiotic and human-gut strains of bifidobacteria (PHBifs); Table S6: A list of the genes and their corresponding functions present in the core-genome of the 56 probiotic and human-gut strains of bifidobacteria (PHBifs); Table S7: The genome-sizes and abundances of the mobile genetic elements present in the genomes of the 56 probiotic and human-gut strains of bifidobacteria (PHBifs); Table S8: The correlation statistics of the mobile genetic elements of the 56 probiotic and human-gut strains of bifidobacteria (PHBifs); Table S9: The correlation statistics of the genome-sizes versus the abundances of COGs classes present in the genomes of the 56 probiotic and human-gut strains of bifidobacteria (PHBifs); Table S10: The KEGG pathway functions (level-I) of the pan-genome of the 56 probiotic and human-gut strains of bifidobacteria (PHBifs); Table S11: The KEGG pathway functions (level-II) of the pan-genome of the 56 probiotic and human-gut strains of bifidobacteria (PHBifs); Table S12: A list of the human proteins and the core proteins of the 56 probiotic and human-gut strains of bifidobacteria (PHBifs) predicted to be interacting with each other; Table S13: Detailed functions of the core proteins of the 56 probiotic and human-gut strains of bifidobacteria (PHBifs) predicted to be interacting with the human proteins; Table S14: A list of the core proteins of the 56 probiotic and human-gut strains of bifidobacteria (PHBifs) interacting with each other; Table S15: A list of the core proteins of the 56 probiotic and human-gut strains of bifidobacteria (PHBifs) predicted to be interacting with the human innate immunity related proteins; Table S16: The KEGG pathway information of the human immune related proteins interacting with the core proteins of the 56 probiotic and human-gut strains of bifidobacteria (PHBifs).
